# A qualitative evaluation of participants experiences of living with back pain, lumbar fusion surgery, and post-operative rehabilitation

**DOI:** 10.1186/s40814-022-01050-y

**Published:** 2022-04-25

**Authors:** James Greenwood, Michael Hurley, Alison McGregor, Orla McCourt, Fiona Jones

**Affiliations:** 1 Internal Box 8, Victor Horsley Department of Neurosurgery, National Hospital of Neurology and Neurosurgery, Queen Square, London, WC1 3BG UK; 2 Faculty of Health and Social Care Sciences, St Georges University of London, 2nd Floor Grosvenor Wing, Cranmer Terrace, London, SW17 0RE UK; 3 Biodynamics Lab, Imperial College London, Charing Cross Hospital, Charing Cross Campus, London, W6 8RP UK; 4 Department of Physiotherapy, University College London Hospital, 235 Euston Road, London, NW1 2BU UK

**Keywords:** Qualitative, Rehabilitation, Lumbar fusion, Mixed-methods, Feasibility, Complex intervention, Physiotherapy, Theoretical modelling

## Abstract

**Background:**

The use of lumbar fusion surgery is increasing in developed economies. High levels of patient dissatisfaction are reported post-operatively. To address this need, we developed a theoretically informed rehabilitation programme for use following lumbar fusion surgery (the REFS programme). We conducted a mixed methods randomised controlled feasibility study (REFS v ‘usual care’). The numerical and feasibility outcomes are reported separately. The current qualitative study was ‘nested’ within the main feasibility study to explore participants’ experiences before and after lumbar fusion surgery including the impact of rehabilitation content. This facilitated a deeper understanding of potential mechanisms of action, for theoretical and programme refinement.

**Methods:**

A purposive sample (*n* = 10 ‘usual care’, *n* = 10 REFS) was identified from the main feasibility study cohort. Individual semi-structured interviews were conducted post-operatively (median 8 months, range 5–11). Interview data were transcribed verbatim, coded, and analysed thematically.

**Results:**

Three themes were constructed: the breadth and severity of impact associated with a chronic lumbar disorder was summarised in theme 1, ‘Ever-decreasing circles; living with a chronic lumbar disorder’. Theme 2, ‘What have I done? Reflections on recovery from lumbar fusion surgery’, illustrated participants post-operative helplessness, which was associated with worsening mental health, problematic use of opioids, fear related to the instillation of metalware, and the important mitigating effect of informal social support. Theme 3 ‘Rehabilitation experiences’ identified critical rehabilitation programme content including exercise, a shared rehabilitation experience, the opportunity for vicarious learning, and professional expertise.

**Conclusions:**

To enhance patient benefit future REFS programme iterations should consider reinforcement of the identified valued programme content. Additional content should be considered to mitigate post-operative fear, which frequently aligned with the instillation of metalware into the spine. Participant’s perceptions regarding the necessity of lumbar fusion surgery has potential implications for the surgical consent process.

**Trial registration:**

Study registration; ISRCTN60891364, date registered 10/7/2014.

**Supplementary Information:**

The online version contains supplementary material available at 10.1186/s40814-022-01050-y.

## Key points

- Participant’s identified valued content of the REFS programme including specific exercises, professional expertise, vicarious learning, social support, education, and a shared rehabilitation environment
- Participants apportioned benefit to a combination of discrete programme elements
- Potentially critical emergent themes were identified including fear related to the instillation of metalware and participants perspectives regarding the necessity for fusion surgery

## Background

The use of lumbar fusion surgery is increasing, particularly in patients over 60 years [1]. In high-income countries, the population of those over is 60 growing faster than any other age group [2]; therefore, the use of fusion surgery is likely to continue, with patients living longer post-operatively. Following lumbar fusion surgery, 40% of patients are unsure or dissatisfied with their outcome [3] reporting impaired psychological, sensory, social, and neuromusculoskeletal function [4].

A recent meta-analysis demonstrated the potential of complex rehabilitation (exercise combined with psychologically mediated content) to improve patient reported outcome following fusion surgery [5]. Currently, no ‘gold standard’ rehabilitation regime exists, and to bridge this research gap, there is an urgent need to develop a rehabilitation programme that is acceptable, safe, and affordable for use following lumbar fusion surgery [5].

**Fig. 1:**
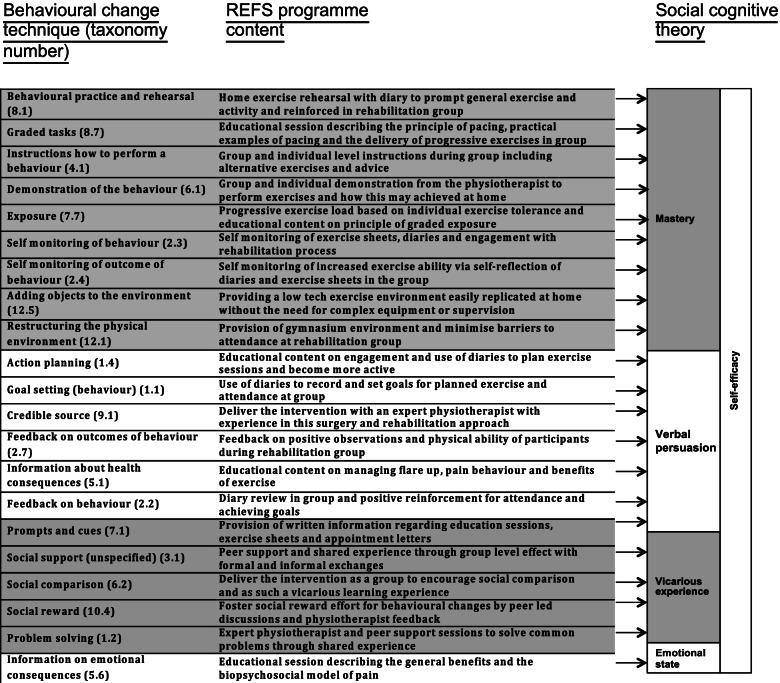
Theoretical framework

Accordingly, we developed the REhabilitation following lumbar Fusion Surgery programme (REFS) utilising the behavioural change wheel methodology [6] and social cognitive theory [7] in keeping with guidance from the Medical Research Council (MRC) [8] (Fig. 1).

This qualitative study is part of a mixed methods feasibility evaluation of the REFS programme, in which participants (*n* = 52) were randomly allocated to either ‘usual care’ or REFS 3 months after lumbar fusion surgery. ‘Usual care’ typically consisted of 6 sessions of individual face-to-face physiotherapy [9]. The REFS programme consisted of ≤ 10 sessions of group based rehabilitation comprising exercise, education, and peer support [10]. Feasibility outcomes including enrolment, engagement, and numerical analyses are described elsewhere [11].

Improved reporting of programmes such as REFS is essential to the advancement of knowledge and programme evaluation [12]. Many rehabilitation outcome research studies have evaluated ‘un-opened’ packages of care in which rehabilitation is evaluated in the aggregate [13]. This ‘black box’ approach to rehabilitation does little to discern valued programme content and identify possible mechanisms of action. The utilisation of qualitative studies to better understand intervention content is recommended by the MRC and the National Institute for Health Research [8, 14]. When qualitative studies are employed, they are frequently used before the trial, in an exploratory manner, rather than after the trial in an evaluative capacity to ‘unpack’ programme content or validate underlying theory [14, 15].

Little is known about patients’ experiences of lumbar fusion surgery; one qualitative study was identified, but this did not consider rehabilitation experiences [4]. Therefore, a gap exists in our understanding of participants experience following lumbar fusion surgery and in particular what aspects of rehabilitation they apportion value to.

### Study aims

The aims of the current qualitative study were:

- Achieve a deeper understanding of the pre- and post-operative experiences of participants undergoing lumbar fusion
- Explore the perceived impact of rehabilitation content to better understand potential mechanisms of action for theoretical and programme refinement

## Methods

### Study design

An open and inductive approach to this qualitative evaluation was adopted, unconstrained by prior assumptions or frameworks. It was anticipated that this approach would better understand participants’ experiences, highlight contextual issues, refine programme theory, and identify emergent themes prior to a future efficacy study. These benefits are particularly relevant to the evolution of a complex programme such as REFS, which comprises several independent and inter-dependant aspects.

### Study settings and recruitment

At the 6-month data collection point in the main feasibility study a brief written narrative in response to the following questions was requested from all participants (*n* = 43).

i) What were the most positive aspects of rehabilitation after your fusion surgery?
ii) What was most difficult about rehabilitation after your fusion surgery?
iii) What would you change about rehabilitation following fusion surgery?

The responses were utilised to identify a purposive sample (*n* = 10 ‘usual care’, *n* = 10 REFS) of those who expressed extreme and midpoint opinions regarding their rehabilitation and develop a topic guide (Additional material 1). The topic guide was refined iteratively throughout the study based upon emerging data and field notes.

Recruitment between September 2015 and Jan 2017 allowed empirical data saturation, with no new descriptive codes, categories, or themes emerging (decided by consensus amongst the research team) [16]. This ensured the study aims were addressed and the full range of experiences was recorded [17]. Study conduct was informed by the consolidated criteria for reporting qualitative research (COREQ) guidelines; a checklist is provided (Additional material 2) [18].

### Data collection

Data were collected via individual semi-structured interviews and recorded. Interviews were transcribed verbatim, the first 3 by the corresponding author and subsequently by a professional transcription service. Checks of 20% of transcripts selected at random (OM) ensured transcripts matched the recorded data and that no data were missed or transcribed incorrectly. All participants were anonymised, transcripts were not returned to participants for comments.

### Data analysis

Interview data were analysed thematically in accordance with the 6-step method described by Braun and Clarke 2006 [19] (Table 1). This identifies, analyses, and reports themes within and across participants’ experiences [19, 22]. Thematic refinement continued throughout analysis and report writing to facilitate the elucidation of descriptive accounts into abstracted themes. This approach is particularly suited to evaluations of clinical practice producing an output which spans clinical and academic disciplines [23] and has been used in similar studies [24].

**Table 1:** Method of data analysis, modified from Braun and Clarke [19]

Steps	Detail and author roles
**1. Familiarisation with the data**	Check accuracy, transcripts read twice with and without concurrent audio file and notes made regarding potential coding ideas (JG)
**2. Generating initial codes**	Inductive, data driven, coding of transcripts, (JG, OM). No interpretation made, initial codes discussed with research team and attached to relevant sections of text to ensure context and facilitate emergence of semantic and latent codes. In cases of ambiguity, audio file was replayed and field notes consulted to better understand context
**3. Searching for themes**	Themes developed inductively to incorporate analysis across interviews, leading to preliminary themes and sub-themes. Hand sorting of individual codes to key areas of interest, produced preliminary thematic map. Iterative review clarified the relationships between themes and sub-themes
**4. Reviewing themes**	Preliminary themes checked (FJ) with text to ensure accuracy, refined by consensus discussion with research team and considered internal homogeneity and external heterogeneity, which shared analogous data whilst retaining independence [17]. Consensus amongst research team resolved incongruous findings (MH, FJ, AM), developed formal themes and sub-themes. Produced thematic map (JG). Employed a 2-phase approach, initially linking original text to themes and subsequently ensuring thematic map accurately reflects entire data set. Cognisant of thematic map 10 transcripts were re-read to ensure refinement process produced themes accurately reflecting data
**5. Defining and naming themes**	Final refinement of themes and sub-themes undertaken (JG) and discussed amongst research team to achieve consensus (MH, FJ, AM). Themes and sub-themes described and named. Achieved an accurate, clear, and balanced interpretation of the data [19–21].
**6. Producing a report**	Select compelling text examples to illustrate themes. Relate analysis to data and literature to produce report (JG)

## Results

### Interview participants

Participants (*n* = 10 REFS group, *n* = 10 ‘usual care’) were purposefully identified from the feasibility cohort (Table 2) and face to face interviews conducted, in the hospital (*n* = 14), over the telephone (*n* = 4), and an alternate location (*n* = 1 school, *n* = 1 hotel). This facilitated access for participants with limited mobility or those who had returned to work. Interviews were conducted post-operatively (median 8 months, range 5–11) producing 240,095 words for analysis.

**Table 2:** Background characteristics of participants and interview duration

Participant ID	Age	Gender	Group (RG or UC)	Duration of interview (hours.min.seconds)
1	50	F	UC	47.51
2	36	F	UC	32.45
3	51	M	RG	33.46
4	52	F	UC	59.49
5	30	F	UC	1.11.22
6	69	F	RG	38.39
7	46	F	UC	44.23
8	32	M	RG	1.11.44
9	73	M	RG	41.20
10	68	F	RG	58.35
11	52	F	UC	50.20
12	62	M	RG	31.13
13	43	F	RG	42.58
14	58	F	UC	35.21
15	50	M	UC	55.01
16	44	F	RG	37.34
17	53	M	UC	27.02
18	51	F	UC	1.11.44
19	66	F	RG	1.04.25
20	41	F	RG	53.05

*RG* REFS group, *UC* “usual care” group

**Fig. 2:**
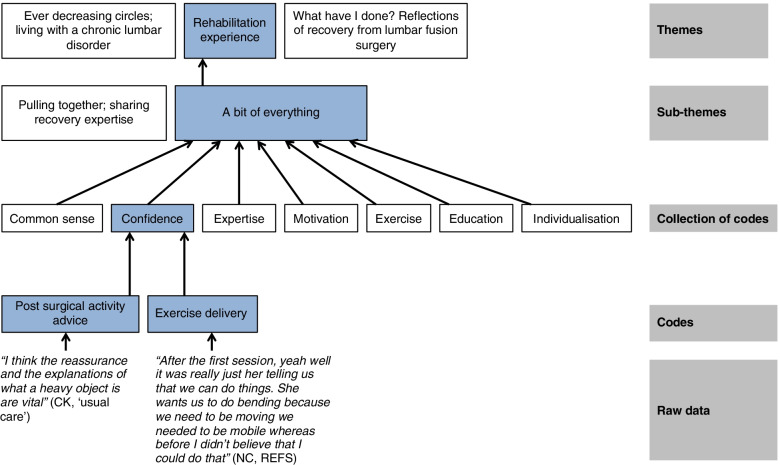
Coding tree

Three themes and 6 sub themes were developed following analysis, an example illustrating the development of ‘Rehabilitation experience’ is presented in the coding tree (Fig. 2).

### Theme 1: Ever decreasing circles, living with a chronic lumbar disorder

**Fig. 3:**
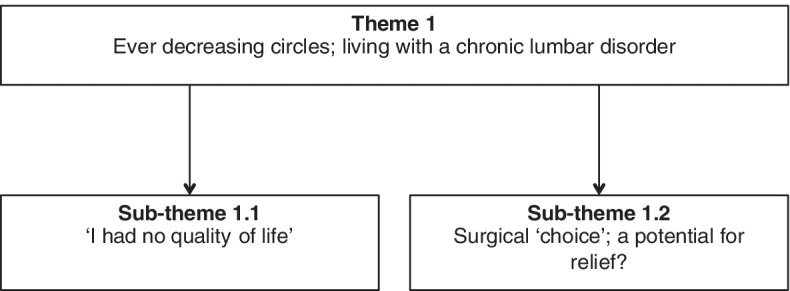
Theme 1 with sub-themes

*‘Ever decreasing circles’* was developed from participant’s descriptions of living with their chronic lumbar disorder, which had a broad impact across physical, psychological, and social domains (Fig. 3).

#### Sub theme 1.1: I had no quality of life

Participants felt the lumbar disorder impaired their quality of life in a variety of ways, including overwhelming pain, and a reduced ability to perform meaningful tasks, socialise, or retain employment status. Such reports highlighted participant’s frustration, frequently aligning their reduced quality of life with impaired mental health.

“the psychological impact of having 6 years, of gradually deteriorating pain and the impact that has on your relationships and your mental wellbeing, the impact that has, I don’t think it can be understated” (EV, REFS).

These frustrations were exacerbated by worries regarding the validity of their condition, financial security, activity planning, or potential future disability. Ultimately, the impact of the lumbar disorder was often associated with an unwelcome change in role.

“I’m not even 60 yet and I don’t want to be this old person sitting in a wheelchair” (GG, ‘usual care’).

#### Sub-theme 1.2: Surgical ‘choice’; a potential for relief?

Most participants were relieved when surgical consultations established an organic basis for their symptoms.

“it was a relief to know that actually there was a real reason for this pain and it wasn’t all in my head” (CK, ‘usual care’).

Participants commonly perceived no viable alternative to lumbar fusion and therefore considered surgery an absolute necessity. The depth of these sentiments were reflected in reports of the surgical consent process, during which discussions describing the risks and potential benefits of fusion surgery were often perceived as irrelevant.

‘Well I just got to believe that I cant live any longer with this, I would go for the surgery no matter what risks there were I would take them” (FG, REFS).

Theme 1 highlights breadth of impact associated with living with a chronic lumbar disorder. Correspondingly, surgical consent discussions were commonly disregarded once an organic basis to the disorder was established.

### Theme 2: ‘What have I done?’ Reflections on recovery from lumbar fusion surgery

**Fig. 4:**
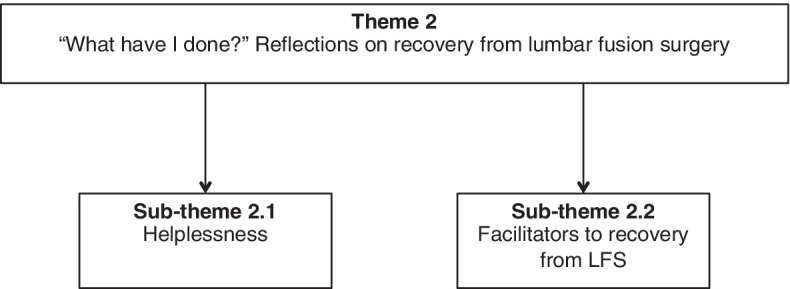
Theme 2 with sub-themes

‘What have I done?’ (Fig. 4) illustrates the heterogeneous response following lumbar fusion surgery and the myriad of factors, which influenced early perceptions of recovery.

#### Sub-theme 2.1: Helplessness

Post-operatively participants often expressed helplessness, which was coupled with their surgical experience. Most were fearful that the surgical implants may move or break and cause them harm. These fears often persisted and limited behaviour such as sexual function and engagement with exercise.

“you know don’t bend or twist and then you’ve got all these images of this metal stuff in your back and what’s going on” (EB, ‘usual care’).

In many instances, the fear of implant failure was exacerbated by the perceived inadequacy of advice.

“I was told to do nothing for 3 months, so I literally take that as literal, do nothing. So lots of bed rest, hardly sitting erm, you know not even being able to help do the dinners or anything like that because I was too scared to lift anything or bend” (NC, REFS).

Other factors consistently associated with post-operative helplessness included a decline in mental health and the use of opioids.

“I was spiralling into it [depression] very, very quick after the operation and that for me, that all came about because of being helpless” (JJ, ‘usual care’).

#### Sub-theme 2.2: Facilitators to recovery from lumbar fusion surgery

Reports across the data identified factors including nursing and surgical input, which participants felt facilitated recovery.

“Hospital was absolutely amazing. I mean if I had any questions the nurses were always there to ask anything or the surgeons came around” (NC, REFS).

Support from family and friends often bridged the gap between hospital and home. This frequently comprised of assistance with simple tasks, such as shopping or collecting medication. Companionship was also valued given the diminished opportunity for social interaction.

Three participants adopted a pragmatic experiential learning approach to recovery, in which activity was guided by personal intuition and symptom response.

“I wasn’t told when I could go walking so I just took it upon myself, thought I can walk a bit better now so I wouldn’t be seen outside with crutches or anything” (GG, ‘usual care’).

Theme 2 illustrates participants’ perceptions of post-operative helplessness, which were particularly aligned with fear related to the introduction of metalware into the spine. The importance of hospital staff and social support networks were identified.

### Theme 3: Rehabilitation experiences

**Fig. 5:**
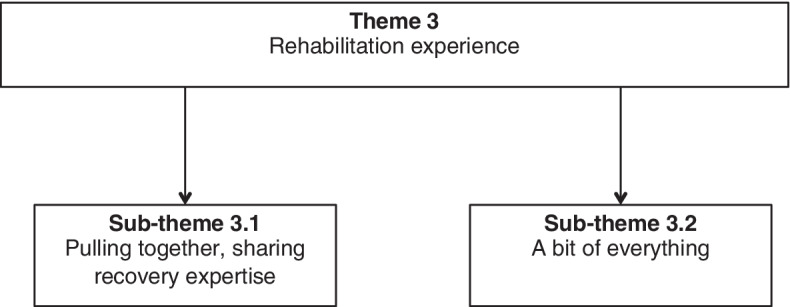
Theme 3 with sub-themes

The theme ‘Rehabilitation experiences’ (Fig. 5) evolved from analysis of data in which participants identified valued rehabilitation aspects. Both sub-themes highlight areas of commonality and divergence across the data, which is occasionally aligned with group allocation (REFS or ‘usual care’). This identifies potential mechanisms of action, facilitates evaluation of the theoretical model, and provides a plausible explanation for the numerical results of the main feasibility study [11].

#### Sub-theme 3.1: Pulling together, sharing recovery expertise

Across the data, participants shared perceptions of the reassurance attributed to professional guidance.

“I was shown and encouraged to do things I wouldn’t have tried on my own, I wouldn’t have done certain exercises or movements, I would have been far too worried that I damaged something or I would do something to myself”, (KC, ‘usual care’).

Participants commonly apportioned value to the physiotherapist’s specific understanding of fusion surgery.

“a trained physiotherapist who knows erm, quite a bit about spinal surgery, so and the safety thing there, you know you feel, you feel quite safe” (EO, REFS).

However, expertise was not exclusively provided from the physiotherapists to the participants. Those allocated to REFS frequently discussed the benefits of sharing their recovery experiences with other participants. These accounts varied in subtle and discrete ways. Many felt observing the rehabilitation progress of others was particularly beneficial.

“it boosts your confidence, you can see what other people are doing and you can think well maybe I’ll have a go of that” (DS, REFS).

Informal discussions with other participants provided the opportunity to exchange recovery perspectives. The shared surgical experience was highly valued and participants emphasised the importance of this mutual understanding, which fostered a positive environment for recovery.

“what you’re getting is access to other people who have had the surgery and they understand you cos I think its really hard for people to understand what you’ve been through” (EV, REFS).

These accounts of a shared rehabilitation experience were generally present in REFS group data as ‘usual care’ was primarily delivered on an individual basis. However, three ‘usual care’ participants experienced group-based rehabilitation, which comprised of mixed patient cohorts (not exclusively lumbar fusion patients) and did not appear to offer the same opportunity for sharing rehabilitation expertise or hold the same meaning for participants.

“I think that would have been great to talk to people with exactly the same experience” (KC, ‘usual care’).

Overall ‘pulling together’ illustrated the value of shared learning through observation, surgical experience, informal exchanges, and group level accountability.

#### Sub-theme 3.2: A bit of everything, combining rehabilitation content

Whilst many participants considered exercise integral to rehabilitation, subtle disparities existed between these reports, which aligned with group allocation.

Participants allocated to ‘usual care’ reported exercise based self-management; however, motivation was a consistent barrier to this rehabilitation approach. Exercise-based self-management also appeared to influence perceptions of recovery with these participants describing their recovery in terms of enhanced physical fitness.

“the main emphasis by then was to strengthen the muscle…..I could see for myself I felt fitter” (KC, ‘usual care’).

Those participants allocated to REFS often discussed valued programme content in addition to exercise, for example, education, which was part of the REFS programme, with allocated time for delivery of pre-planned content.

“having the little talk beforehand and it gives you confidence because you know like the flare up for instance, I thought oh it can happen to other people, but if I was at home on my own without sort of doing my little exercises I would be worrying” (EO, REFS).

This combined approach to group-based rehabilitation altered the manner in which REFS participants perceived their recovery, frequently reporting enhanced confidence, motivation, or reduced fear.

“I think that is such a big part of what the course achieved for me was the, to eliminate the fear of doing things and the fear of recovery because actually the recovery is linked to doing things” (EV, REFS).

Such experiential nuances in rehabilitation content may have important implications, providing potential mechanisms of action to explain the positive clinical impact of REFS. When asked to identify preeminent programme content, participants felt they could not. Without exception, they valued the combination of content.

“It’s a bit of all of it” (FG, REFS).

Theme 3 highlights the range of factors influencing perceptions of rehabilitation, which frequently aligned with group allocation. Participants from both groups valued the professional guidance of a physiotherapist. Those allocated to REFS particularly valued the combination of rehabilitation content, primarily the opportunity for sharing recovery experiences, the physiotherapist’s surgical expertise, and the provision of specific educational content and safe exercises.

## Discussion

This study reinforces guidance from the MRC demonstrating how reliance on numerical outcomes for the evaluation of complex interventions may underreport key areas of value and fail to identify new and potentially critical intervention aspects [8]. The findings illustrate the complexity of participants needs following lumbar fusion and highlight the potential limitations of current exercise-based self-management strategies.

The sub-theme ‘I had no quality of life’ highlights participants progressive pre-operative decline in physical, mental, and social function. This shares considerable overlap with the abstracted theme ‘my life is impoverished and confined’ from a recent mega-ethnographic review of patients with chronic non-malignant pain [25]. The breadth of this pre-operative impact likely contributes to the heterogeneous response to fusion surgery and the complexity of reported post-operative needs [4].

To address this complex post-operative need, valued rehabilitation aspects were identified. Across the data, exercise and professional support were perceived as central components of rehabilitation. Additionally, REFS participants considered the provision of formal education, an opportunity for social exchange, and vicarious learning as important. Systematic reviews across a range of long-term conditions have demonstrated the benefit of peer support in group-based rehabilitation [26–28]. Therefore, the sub-theme ‘Pulling together, sharing recovery expertise’ represents a plausible mechanism of action explaining the favourable numerical outcomes of REFS compared with ‘usual care’ [11].

Whilst REFS participants could describe discrete valued programme content (e.g. exercise, professional guidance, shared rehabilitation expertise), they were unable to identify preeminent aspects. This is likely explained by the independent and inter-dependant nature of complex programme content. The combination of active components is a recognised feature of complex interventions [8] and supports the development of the sub-theme ‘A bit of everything’.

The REFS programme was informed by the social cognitive theory in which selected behavioural change techniques were mapped to components of self-efficacy (mastery, verbal persuasion, vicarious observation, emotional state) (Fig. 1). Enhancing self-efficacy has been positively associated with recovery from diverse conditions including orthopaedic trauma and stroke [29, 30]. The results of the current study support the adoption of the social cognitive theory as an overarching programme theory. When considered at the level of discrete behavioural change techniques (taxonomy number), qualitative evidence was identified supporting the inclusion of graded tasks (8.7), instructions on how to perform a behaviour (4.1), demonstration of the behaviour (6.1), exposure (7.7) adding objects to the environment (12.5), credible source (9.1), information about health consequences (5.1), social support (unspecified) (3.1), social comparison (6.2), and social reward (10.4). It is likely that this combined valued programme content contributes to the active mechanisms by which REFS achieved a favourable outcome over ‘usual care’ [11].

A previously unreported finding of this study is the association between a fear of movement and the surgical introduction of metalware to the spine. Kinesiophobia following lumbar fusion surgery has been described [31, 32], but the contributing factors have not been identified. This was illustrated in the sub-theme ‘Helplessness’, which is important as helplessness is associated with post-operative satisfaction [33], and high levels of patient dissatisfaction are reported after lumbar fusion surgery [34, 35].

Sub-theme 1.2 ‘Surgical ‘choice’: a potential for relief’ highlights the perceived essential nature of fusion surgery and participants’ potential indifference to the risks of spinal fusion. Clinical teams delivering spinal fusion surgery should consider the findings of this study when requesting consent. Although guidance exists regarding consent [36], surgical teams should consider the potential for considerable divergence in the perceived necessity for spinal fusion between themselves and their patients. Post-operatively the provision of activity guidance should be clearer and formal rehabilitation should be considered as suggested in a recent meta-analysis [5].

The findings of this qualitative study should be combined with the numerical results to refine the REFS programme theory and consider the implications for revised programme content and delivery. Future research should consider an adequately powered study evaluating the efficacy of a revised REFS programme. This should include mixed methods with planned mediation analysis to better evaluate potential mechanisms of action.

## Conclusions

Future REFS programme iterations should include reinforcement of the valued content identified in this study and consider the inclusion of new specific content to address kinesiophobia related to the surgical introduction of metalware into the spine. A future REFS efficacy study should consider utilising mediation analysis to better evaluate the impact of discrete programme aspects.

Previous reports describe patients making balanced decisions regarding spinal surgery, based on the severity and duration of pain, and walking impairment [37]. Our results do not support this. The perceptual gap between participants and surgical teams regarding the necessity for lumbar fusion warrants further exploration.

### Study limitations

Conceivably, the interviewers’ prior assumptions influenced the data collection and analysis. To reduce this potential bias, a third party interviewer (OM), cross checking codes, and thematic refinement across the research team were employed.

## Supplementary Information

**Additional file 1: Additional material 1.** Topic guide.

**Additional file 2: Additional material 2.** COREQ checklist.

## Data Availability

The datasets generated and analysed during the current study are not publicly available due to ethical constraints but are available from the corresponding author on reasonable request.

## Abbreviations

REFS: Rehabilitation following lumbar fusion surgery
MRC: Medical Research Council
