# Supplementary material for: A qualitative evaluation of participants experiences of living with back pain, lumbar fusion surgery, and post-operative rehabilitation
Source: Pilot Feasibility Stud. 2022 Apr 25;8:91. doi: 10.1186/s40814-022-01050-y (PMC9036810; doi:10.1186/s40814-022-01050-y)
Supplement: Supplementary file 1 — Additional file 1: Additional material 1. Topic guide. [file 40814_2022_1050_MOESM1_ESM.docx]

**Additional material**

**1-Topic guide.**

**Exploring the experience of rehabilitation following lumbar fusion surgery.**

**1-Introduction**

- Introduce self and why we are asking questions, who is funding this and why the pt has been selected from the study cohort.
- Explain about recording device and transcription process.
- How long will the interview go on for, (max 1 hour).
- Payment for travel reimbursement arrangement.
- Ethical considerations eg; right to withdraw, what will happen to audio recording, confidentiality of data, dissemination plan.
- Any questions that the participant may have/reassure participant that this is just a way of understanding their experiences.

**2-Background information**

2.1 I am interested in how back pain affected your life before surgery. Can you tell me a bit about this, what was the impact of back pain on your life?

***Prompts***

- ***That’s interesting, can you tell me a little more about that?***
- ***Family, social, work, sleep etc***

2.2 Most people have tried other treatments before considering surgery. Could you just tell me a little about the sort of treatments that you tried before deciding on fusion surgery?

***Prompts***

- ***How would you say you were coping at this time?***
- ***What was your understanding about your back?***
- ***What were your thoughts/feelings about you condition?***
  - - ***Why?***
    - ***How did that make you feel?***

**3-In patient / early post op care.**

3.1 I would like to know your thoughts on the surgery and the hospital stay.

What stood out for you and what do you remember about your stay?

- ***What was good or bad about being in hospital?***
  - - ***Do you know why those things were important to you?***
    - ***Was there anything else?***

3.2 Could you tell me something about the first few weeks/months after your surgery? What can you remember about coping at home?

***Prompts***

- ***What were the main issues you had to deal with?***
  - - ***Could you tell me a little more about that?***
    - ***Were there any other things that you felt were important at this time?***
    - ***Why was that?***
- ***Would it be fair to summarise? Is there anything you would like to add?***
  - - ***Why do you think you say that?***

**4-Rehabilitation.**

4.1 Now I would like to ask you about the rehabilitation that you received, were you in the RG or UC group?

4.2 Can you tell me about your experiences of rehabilitation?

***Prompts***

- ***Some people remember different aspects, were there any aspects (good or bad) that were of specific importance or memorable to you?***
  - - ***If so what?***
    - ***How did these things help or hinder you?***
    - ***Why do you think that was the case?***
  1. I am interested in exploring your thoughts about your rehab particularly what impact did it have if any on your recovery – can you tell me a little more/give some examples?

***Prompts***

- ***Can you give me some examples of which aspects of the rehabilitation were most important to you?***
  - - ***Why do you think that is?***
    - ***Was that helpful?***
    - ***Is there anything that could be done to make the most of these aspects?***
- ***Could you give me some examples of the aspects that you found difficult/unhelpful about the rehab?***
  - - ***What do you think should be changed?***
- ***What would you say to a friend who asked you how it had gone?***
  - - ***Why do you think that is?***
    - ***Could you tell me more please?***
- ***If I can summarise, does this sound like your perspective? Would you like to add anything?***
  - - ***Why do you feel these things are important?***
- ***What will you be doing to look after your back in the future?***
  - - ***How do you think your perspectives compare between now and before surgery?***

**5-Longer term/satisfaction.**

5.1 Thinking about the future now, how do you think your back will affect you in the next few years, do you feel positive? Do you think your back will be having much of an impact?

***Prompts***

- ***If a friend asked you to summarise your back operation what would you say?***
- ***Could you give me an example of what do you feel were the most difficult and best aspects of the experience?***
  - - ***What could have been done differently?***
    - ***Why do you think that would be important?***
- ***What do you think your future holds regarding your back?***
  - - ***For example do you think you will cope?***
    - ***Why do you think that?***
    - ***What could have been done?***

5.3 Do you have anything else you would like to tell us?

Thank you very much for helping with this, it is very useful in helping us to understand what is important to patients. This will help us shape the way we develop our approach to rehabilitation and ensure that what matters to the patient is upper most in our thoughts.
