# Supplementary material for: A qualitative evaluation of participants experiences of living with back pain, lumbar fusion surgery, and post-operative rehabilitation
Source: Pilot Feasibility Stud. 2022 Apr 25;8:91. doi: 10.1186/s40814-022-01050-y (PMC9036810; doi:10.1186/s40814-022-01050-y)
Supplement: Supplementary file 2 — Additional file 2: Additional material 2. COREQ checklist. [file 40814_2022_1050_MOESM2_ESM.docx]

**Additional material**

**2-COREQ checklist.**

| **Domain 1: Research team and reflexivity** | | |
| --- | --- | --- |
| **1** | Interviewer | JG, OM |
| **2** | Credentials | JG=MRes, PhD student, OM=BSc (hons) |
| **3** | Occupation | JG=ESP physio, OM=Specialist physio |
| **4** | Gender | JG=male, OM=female |
| **5** | Experience and training | JG=mentoring, short courses, self directed reading, faculty presentations. OM=previous research assistance in qualitative studies |
| Relationship with participants | | |
| **6** | Relationship established | JG=Chief investigator and responsible for recruitment (known to participants). OM=unknown to participants. |
| **7** | Participant knowledge of the interviewer | All participant knew the role of JG in the research. OM had no direct involvement in the study beyond conduct of the interview. |
| **8** | Interviewer characteristics | Bias is largely through development of theoretical framework and REFS (JG). |
| **Domain 2: Study design** | | |
| Theoretical framework | | |
| **9** | Methodological orientation and theory | Thematic analysis |
| Participant selection | | |
| **10** | Sampling | Purposive |
| **11** | Method of approach | Face to face and telephone |
| **12** | Sample size | 20 |
| **13** | Non-participation | 6 |
| Setting | | |
| **14** | Setting of data collection | Hospital (non-clinical) and other as described |
| **15** | Presence of non-participants | No |
| **16** | Description of sample | Based on response to commentary narrative |
| Data collection | | |
| **17** | Interview guide | Provided with prompts, developed iteratively |
| **18** | Repeat interviews | No |
| **19** | Recording | Digital audio |
| **20** | Field notes | Yes, after in reflexive diary and prior in field notes |
| **21** | Duration | Approximately 60 minutes |
| **22** | Data saturation | Yes no new themes emerged |
| **23** | Transcripts returned | No |
| **Domain 3: Analysis and findings** | | |
| Data analysis | | |
| **24** | Number of coders | 3 |
| **25** | Coding tree | Yes provided |
| **26** | Derivation of themes | Inductive |
| **27** | Software | No |
| **28** | Participant checking | No |
| Reporting | | |
| **29** | Quotations presented | Yes |
| **30** | Data and findings consistent | Yes |
| **31** | Clarity of major themes | Yes |
| **32** | Clarity of minor themes | Yes |
